# Two new species and one new provincial record of *Cantharellus* (*Hydnaceae*, *Cantharellales*) from the Dabie Mountains, China

**DOI:** 10.3897/mycokeys.141.210171

**Published:** 2026-10-01

**Authors:** Hang Chu, Yong-lan Tuo, Bo Zhang, Xiao Li

**Affiliations:** 1 Sanjiang Fungal Industry Collaborative Innovation Center, Jilin Agricultural University, Changchun 130118, China Sanjiang Fungal Industry Collaborative Innovation Center, Jilin Agricultural University Changchun China; 2 Engineering Research Center of Edible and Medicinal Fungi Ministry of Education, Jilin Agricultural University, Changchun, 130118, China Engineering Research Center of Edible and Medicinal Fungi Ministry of Education, Jilin Agricultural University Changchun China; 3 Industrial Development Institute for Plants, Animals and Fungi Integration of Biyang County, Biyang 463799, China Industrial Development Institute for Plants, Animals and Fungi Integration of Biyang County Biyang China

**Keywords:** Chanterelle, novel taxa, phylogeny, taxonomy

## Abstract

*Cantharellus* is a genus of *Hydnaceae (Cantharellales)* with significant ecological and economic importance; however, its species diversity in central China remains largely unexplored. In this study, 16 specimens of *Cantharellus* were collected from the Dabie Mountains, China. Based on morphological observations and multigene phylogenetic analyses of nrLSU, *rpb2*, and *tef1-α*, two new species, *C. armeniacus* and *C. cremeus*, are described. Additionally, *C. curvatus* is reported as a new record for Anhui Province, representing a range extension for this species, and the occurrence of *C. phloginus* in the region is documented. Detailed macroscopic and microscopic descriptions, illustrations, and taxonomic discussions are provided, expanding current knowledge of the diversity of *Cantharellus*.

## Introduction

*Cantharellus* Adans. ex Fr. belongs to the *Basidiomycota*, *Agaricomycetes*, *Cantharellales*, and *Hydnaceae* (Cao et al. 2021). Several species of this genus, such as *C. cibarius* Fr. and *C. alborufescens* (Malençon) Papetti & S. Alberti, are highly valued for their nutritional composition and reported medicinal properties (Ge et al. 2023; Moghaddam et al. 2023; Chen and Xu 2024; Kumar et al. 2025). Species of *Cantharellus* are ectomycorrhizal fungi that form symbiotic associations with various host plants (Pilz et al. 2003; Yun and Hall 2004), including members of *Fagaceae* and *Pinaceae* (Buyck et al. 2014), thereby playing a crucial role in forest ecosystems.

Most *Cantharellus* species produce brightly colored basidiomata characterized by lamellate or nearly smooth hymenophores and smooth basidiospores (Wilson et al. 2012; Buyck et al. 2014; Zhang et al. 2021). Although these species are readily identifiable at the generic level in the field, they exhibit considerable morphological similarity and extensive intraspecific variation. Consequently, species identification based solely on morphological characters is often unreliable, resulting in species misidentification, an underestimation of species diversity within the genus, and an incomplete understanding of distribution patterns. The use of DNA markers has proven effective in facilitating species delimitation in *Cantharellus*, particularly for taxa that are challenging to distinguish based on morphology alone, and continues to refine the taxonomic framework of this genus (Buyck et al. 2014, 2016; Shao et al. 2016; Zhang et al. 2021; Zhao et al. 2021; Wang et al. 2023).

Species of the genus *Cantharellus* are distributed worldwide, and the genus exhibits particularly high diversity in subtropical to tropical regions (Buyck et al. 2014). Phylogenetic analyses based on multigene datasets have recognized seven subgenera within *Cantharellus* (Buyck et al. 2014; Cao et al. 2021), comprising a total of 257 species (Westrip et al. 2026). In recent years, Asia, and especially China, has become a focal region for research on *Cantharellus* diversity. Based on integrated morphological and molecular phylogenetic analyses, numerous new species from Asia have been identified and described (Shao et al. 2016; An et al. 2017; Antonín et al. 2017; Ogawa et al. 2018; Jian et al. 2020; Zhang et al. 2025). Currently, five subgenera and 43 species of *Cantharellus* are recognized in China, as summarized in Table 1 (Wang et al. 2023, 2025; Zhang et al. 2025; Huang et al. 2026).

**Table 1.** Reported *Cantharellus* species in China.

**Subgenus Afrocantharellus**		**Subgenus *Cantharellus***	**Subgenus *Cinnabarinus***	**Subgenus *Parvocantharellus***	**Subgenus *Magni***
*C. cineraceus*	*C. albopileatus*	*C. danxiashanensis*	*C. albidohymenius*	*C. bellus*	
*C. hygrophoroides*	*C. chuiweifanii*	*C. chrysanthemoides*	*C. albus*	*C. magnus*	
	*C. cibarius*	*C. chrysanthus*	*C. aurantinus*	= C. laevigatus	
	*C. hainanensis*	*C. citrinus*	*C. austrosinensis*		
	*C. laevihymeninus*	*C. sinocinnabarinus*	*C. convexus*		
	*C. macrocarpus*	*C. phloginus*	*C. curvatus*		
	*C. pseudohainanensis*	= C. albovenosus	*C. galbanus*		
	*C. pinetorus*		*C. koreanus*		
	*C. ravus*		*C. luteovirens*		
	*C. sinolateritius*		*C. minioalbus*		
	*C. subvaginatus*		*C. neopersicinus*		
	*C. tuberculosporus*		*C. pseudoaustrosinensis*		
	*C. vaginatus*		*C. roseocastaneus*		
	*C. versicolor*		*C. subtilis*		
	*C. multilobatus*		*C. parvoluteolus*		
	*C. applanatus*		= *C. luteolus*		
	= C. anzutake		*C. elongatipes*		
	= C. natarajanii		= C. subminor		
			= C. sinominor		
			*C. zangii*		
			= C. sikkimensis		

Note: The species list and nomenclature follow Wang et al. (2023, 2025), Zhang et al. (2025), and Huang et al. (2026). Species names currently treated as synonyms are listed under their accepted names and indicated with “=.”

Among the Chinese species of *Cantharellus*, the diversity within subg. *Parvocantharellus* Eyssart. & Buyck and subg. *Cinnabarinus* Buyck & V. Hofst. is particularly notable. Prior to this study, 17 species of subg. *Parvocantharellus* and six species of subg. *Cinnabarinus* had been identified in China, representing approximately 58.6% and 28.6%, respectively, of the global totals for these subgenera. The distribution of these two subgenera in China is predominantly concentrated in the tropical and subtropical regions of southern China, encompassing 15 provinces and autonomous regions. Guangdong, Yunnan, and Zhejiang provinces exhibit the highest species richness, with 12, nine, and seven species recorded, respectively.

In this study, *Cantharellus* specimens were collected from the Dabie Mountains. The Dabie Mountains are located in central China and extend across Anhui, Hubei, and Henan (Fan et al. 2024). The area encompasses several national nature reserves and exhibits exceptionally high biodiversity (Zhou 2025). In recent years, numerous new fungal species have been discovered in this region (Shen et al. 2025; Song et al. 2026; Tuo et al. 2026). Ten species of *Cantharellus* have been documented in the area (Zhang et al. 2025; Huang et al. 2026). Nevertheless, comprehensive systematic studies focusing on this genus are scarce.

Through morphological examination and multigene phylogenetic analyses, four species belonging to subg. *Cinnabarinus* and subg. *Parvocantharellus* were identified. Among these, two species are new to science: *C. armeniacus* and *C. cremeus*. Furthermore, one species, *C. curvatus* Buyck, R. Ryoo & Antonín, represents a new record for Anhui Province, and the remaining species is the previously reported *C. phloginus* S.C. Shao & P.G. Liu. These findings improve the understanding of species diversity and geographic distribution patterns of *Cantharellus* in China and help clarify the phylogenetic relationships within the genus.

## Materials and methods

### Specimen collection

Field investigations were carried out from July to October in both 2023 and 2024 in the Dabie Mountains, Anhui Province, China. During these investigations, a total of 16 *Cantharellus* specimens belonging to subg. *Parvocantharellus* and subg. *Cinnabarinus* were collected. Detailed observations and documentation of fresh macromorphological characteristics were made prior to drying the specimens in an oven at 45 °C. All voucher specimens were subsequently deposited in the Fungarium of Jilin Agricultural University (**FJAU**).

### Morphological studies

Macromorphological characteristics and habitat descriptions were derived from field notes and photographic documentation, with color references standardized according to the Methuen Handbook of Color (Kornerup and Wanscher 1978). Microscopic observations and measurements were conducted on dried tissue sections mounted in either 5% aqueous potassium hydroxide (KOH) or 1% ammoniacal Congo red to facilitate tissue clearing and mounting. These observations were carried out under a Carl Zeiss Axio Lab A1 compound microscope (Carl Zeiss AG, Jena, Germany) at magnifications up to 1000×. Melzer’s reagent was applied to detect amyloid reactions during microscopic examination. Basidiospore dimensions are reported in the format (a–) b–c (–d), where the range b–c encompasses 90% of the measured values, and a and d represent the minimum and maximum values, respectively. Lm and Wm denote the mean length and width (± SD), respectively; Q indicates the length/width ratio of an individual spore, and Qm represents the mean Q value (± SD). All line drawings of microscopic structures were hand-drawn based on rehydrated specimens.

### DNA extraction, PCR, and sequencing

Genomic DNA was extracted from voucher specimens using the NuClear Plant Genomic DNA Kit (CoWin Biotech Co., Ltd., Jiangsu, China), according to the manufacturer’s protocol. Three genes, including the large subunit of the nuclear ribosomal RNA gene (nrLSU), the translation elongation factor 1-alpha gene (*tef1-α*), and the second-largest subunit of RNA polymerase II (*rpb2*), were selected for PCR amplification and sequencing. The primer pairs LROR/LR5 (Vilgalys and Hester 1990), *tef1F/tef1R* (Morehouse et al. 2003), and RPB2-5FCanth/RPB2-7cRCanth (Buyck et al. 2014) were used to amplify nrLSU, *tef1-α*, and *rpb2*, respectively. PCR reactions were performed in a 25 μL volume containing 1 μL of genomic DNA template, 14 μL of ddH_2_O, 1 μL each of forward and reverse primers (10 μM), and 8 μL of SanTaq PCR Master Mix (Sangon Biotech, Shanghai, China). The amplification parameters were set as follows: initial denaturation at 95 °C for 4 min, followed by 35 cycles of denaturation at 94 °C for 60 s, annealing at 53 °C (nrLSU), 50 °C (*tef1-α*), and 52 °C (*rpb2*) for 60 s, and extension at 72 °C for 60 s, with a final extension at 72 °C for 8 min. Amplified products were electrophoresed on 1% agarose gels with standard DNA markers. Sanger sequencing was performed by Comate Bioscience Co., Ltd. (Jilin, China) using the same primers. Raw sequences were assembled with SeqMan (DNASTAR, v7.1). The newly generated sequences were deposited in the National Center for Biotechnology Information (NCBI) database (Sayers et al. 2024).

### Phylogenetic analyses

The dataset used for inferring the multigene phylogeny (nrLSU–*tef1-α*–*rpb2*) was composed of previously published sequences, including those derived from type specimens (Buyck et al. 2014; Buyck 2016; Olariaga et al. 2017; Zhang et al. 2021; Wang et al. 2023; Zhang et al. 2025), together with newly generated sequences from the present study. *Cantharellus cibarius* was designated as the outgroup (Zhang et al. 2022). Detailed information regarding these sequences is summarized in Table 2.

**Table 2.** Specimen information and sequence data used in the phylogenetic analyses. Newly generated sequences from this study are highlighted in bold. Type specimens are indicated as follows: HT, holotype; ET, epitype; PT, paratype; NT, neotype; and “–” denotes unavailable data.

**Species**	**Voucher**	**Locality**	**GenBank accession no**.			**References**
			**nrLSU**	***tef*1-**α	***rpb*2**	
*Cantharellus albidohymenius*	GDGM90220	China	PX524025	PX704885	–	Zhang et al. 2025
*C. albidohymenius*	GDGM90302 (HT)	China	PX524027	–	–	Zhang et al. 2025
*C. afrocibarius*	BB 96. 235 (HT)	Zambia	KF294668	JX192993	KF294746	Buyck et al. 2014
*C. afrocibarius*	BB 96. 236	Zambia	KF294669	JX192994	KF294747	Buyck et al. 2014
*C. albus*	GDGM81399	China	MZ605074	MZ613977	MZ614022	Zhang et al. 2021
*C. albus*	HKAS:107045 (HT)	China	MT782540	MT776015	MT776012	Jian et al. 2020
*C. appalachiensis*	BB 07.123	USA	KF294635	GQ914979	KF294711	Buyck et al. 2011a, 2014
*C. appalachiensis*	JJ MO-cant-3	USA	KX857090	KX857032	KX856994	Buyck 2016
***C. armeniacus***	**FJAU81688 (PT)**	**China**	**PZ379274**	**PZ435484**	**PZ435493**	**Present study**
***C. armeniacus***	**FJAU81689 (HT)**	**China**	**PZ379275**	**PZ426409**	**PZ435494**	**Present study**
***C. armeniacus***	**FJAU81690**	**China**	**PZ379276**	**PZ435485**	**PZ435495**	**Present study**
*C. aurantinus*	GDGM46278 (HT)	China	MZ766517	MZ766560	–	Zhang et al. 2021
*C. aurantinus*	GDGM81889	China	MZ766519	MZ766562	MZ766574	Zhang et al. 2021
*C. austrosinensis*	GDGM81249 (HT)	China	MZ605082	MZ613983	MZ614027	Zhang et al. 2021
*C. austrosinensis*	GDGM82877	China	MZ605088	MZ613990	MZ614033	Zhang et al. 2021
*C. avellaneus*	1217/ER (ET)	Madagascar	KX857093	–	KX856997	Buyck 2016
*C. chrysanthemoides*	GDGM60334	China	PX664379	–	–	Zhang et al. 2025
*C. chrysanthemoides*	GDGM80391 (HT)	China	PX524020	PX704932	PX516759	Zhang et al. 2025
*C. chrysanthus*	GDGM80220 (HT)	China	OM978970	ON119083	ON119019	Zhang et al. 2022
*C. chrysanthus*	GDGM80436	China	OM978971	ON119084	ON119020	Zhang et al. 2022
*C. cibarius*	479/GE 07.025	France	KF294658	GQ914949	KF294736	Buyck and Hofstetter 2011b; Buyck et al. 2014
*C. cibarius*	351/BB 07.300	Slovakia	KF294641	GQ914950	KF294718	Buyck and Hofstetter 2011b; Buyck et al. 2014
*C. cinnabarinus*	BB 07.001 (NT)	USA	KF294624	GQ914985	KF294698	Buyck et al. 2011a, 2014
*C. cinnabarinus*	BB 07.053	USA	KF294630	GQ914984	KF294705	Buyck et al. 2011a, 2014
*C. citrinus*	1691 (HT)	Korea	–	MW124385	MW286818	Buyck et al. 2020a, 2021
*C. citrinus*	1711	Korea	–	MW124384	MW286815	Buyck et al. 2020a, 2021
*C. coccolobae*	1064/RC 14.24	Guadeloupe	KX857088	KX857020	KX856992	Buyck 2016
*C. coccolobae*	1065/RC 11.25 (HT)	Guadeloupe	KX857089	KX857021	KX856993	Buyck 2016
*C. congolensis*	1645/BB 16.044	Central African Republic	KX857102	KX857075	–	Buyck 2016
*C.* aff*. congolensis*	BB 06.176	Madagascar	KF294606	JX192967	KF294680	Buyck et al. 2014
C. aff. congolensis	BB 06.197	Madagascar	KF294608	GQ914982	KF294683	Buyck et al. 2014
*C. convexus*	GDGM54841	China	OM978940	ON119052	ON119036	Zhang et al. 2022
*C. convexus*	GDGM60307 (HT)	China	OM978941	ON119053	ON119037	Zhang et al. 2022
*C. corallinus*	1083/JJ MO/CANT-2 (HT)	USA	KX896776	KX857031	ON260862	Buyck 2016; Wang et al. 2023
*C. corallinus*	1086/JJ MO/CANT-5	USA	–	KX857034	ON260863	Buyck 2016; Wang et al. 2023
***C. cremeus***	**FJAU81685**	**China**	**PZ379271**	**PZ426406**	**PZ435490**	**Present study**
***C. cremeus***	**FJAU81686 (PT)**	**China**	**PZ379272**	**PZ426407**	**PZ435491**	**Present study**
***C. cremeus***	**FJAU81687**	**China**	**PZ379273**	**PZ426408**	**PZ435492**	**Present study**
***C. cremeus***	**FJAU81691**	**China**	**PZ379277**	**PZ426410**	**PZ435496**	**Present study**
***C. cremeus***	**FJAU81692**	**China**	**PZ379278**	**PZ426411**	**PZ435497**	**Present study**
***C. cremeus***	**FJAU81693 (HT)**	**China**	**PZ379279**	**PZ426413**	**PZ435498**	**Present study**
***C. cremeus***	**FJAU81694**	**China**	**PZ379280**	**PZ426414**	**PZ435499**	**Present study**
***C. cremeus***	**FJAU81695**	**China**	**PZ379281**	**PZ426415**	**PZ435500**	**Present study**
*C. curvatus*	1695/VA14.57 (PC0142461) (HT)	Korea	–	MW124390	–	Buyck et al. 2020a
*C. curvatus*	XHW2590 (KUN-HKAS 58235)	China	KU720331	KM893840	–	Wang et al. 2023
***C. curvatus***	**FJAU81696**	**China**	**PZ379282**	**PZ435486**	**PZ435501**	**Present study**
***C. curvatus***	**FJAU81697**	**China**	**PZ379283**	**PZ426412**	**PZ435502**	**Present study**
***C. curvatus***	**FJAU81698**	**China**	**PZ379284**	**PZ435487**	**PZ435503**	**Present study**
*C. cyphelloides*	TNS:F-61721 (HT)	Japan	LC047741	–	–	Suhara and Kurogi 2015
*C. cyphelloides*	TNS:F-61726	Japan	LC047742	–	–	Suhara and Kurogi 2015
*C. danxiashanensis*	GDGM77227 (HT)	China	PX524022	PX704952	–	Zhang et al. 2025
*C. danxiashanensis*	GDGM77228	China	PX524023	PX704953	–	Zhang et al. 2025
*C. decolorans*	BB 08.278 (ET)	Madagascar	KF294654	GQ914968	KF294731	Buyck et al. 2011a, 2014
C. aff. decolorans	BB 06.145	Madagascar	–	JX193003	–	Buyck et al. 2011a
C. aff. decolorans	BB 06.146	Madagascar	KF294603	JX192964	KF294677	Buyck et al. 2014
*C. diminutivus*	DS 06.033	Malaysia	KF294661	–	KF294740	Buyck et al. 2014
*C. elongatipes*	HKAS 110274	China	–	ON089624	ON089633	Wang et al. 2023
*C. elongatipes*	HKAS 110275	China	ON074610	–	ON089634	Wang et al. 2023
*C. friesii*	1001/EC 09.16	Italy	KX857083	KX857015	KX856987	Buyck 2016
*C. friesii*	AH44798	Spain	KR677522	KX828831	KX828752	Olariaga et al. 2015, 2017
*C. galbanus*	GDGM86249 (HT)	China	–	MZ766569	MZ766577	Zhang et al. 2021
*C. garnierii*	1020/BB 09.024	New Caledonia	KX857085	KX857017	KX856989	Buyck 2016
*C. garnierii*	1021/BB 09.033	New Caledonia	KX857086	KX857018	KX856990	Buyck 2016
*C. garnierii*	1030/BB 09.283	New Caledonia	KX857087	KX857019	KX856991	Buyck 2016
*C. koreanus*	1689/VA 13.136 (HT)	Korea	–	KY271941	ON260857	Antonín et al. 2017; Wang et al. 2023
*C. koreanus*	1697/VA 14.115	Korea	–	KY271940	–	Antonín et al. 2017
*C. littoralis*	1007/E.Randrianjohany 107	Madagascar	MT006311	MT002279	MT002275	Buyck et al. 2020b
*C. littoralis*	1008/E.Randrianjohany 2 (HT)	Madagascar	MT006312	MT002280	MT002276	Buyck et al. 2020b
*C. luteolus*	GDGM44258	China	MZ766514	MZ766566	MZ766570	Zhang et al. 2021
*C. luteolus*	GDGM60393 (HT)	China	MZ766515	MZ766568	MZ766575	Zhang et al. 2021
*C. luteolus*	GDGM86247	China	MZ766513	MZ766567	MZ766576	Zhang et al. 2021
*C. luteovirens*	GDGM80672 (HT)	China	MZ605090	MZ613992	MZ614035	Zhang et al. 2021
*C. luteovirens*	GDGM81079	China	MZ605092	MZ613994	MZ614036	Zhang et al. 2021
*C. minioalbus*	GDGM78901 (HT)	China	MZ605097	MZ613998	MZ614042	Zhang et al. 2021
*C. minioalbus*	GDGM78916	China	MZ605100	MZ614001	MZ614045	Zhang et al. 2021
*C. minor*	BB 07.002	USA	KF294625	JX192978	KF294699	Buyck et al. 2014
*C. minor*	BB 07.057	USA	KF294632	JX192979	KF294707	Buyck et al. 2014
*C. neopersicinus*	GDGM85145	China	–	ON119056	ON119041	Zhang et al. 2022
*C. neopersicinus*	GDGM87366 (HT)	China	OM978943	ON119057	ON119042	Zhang et al. 2022
*C. neopersicinus*	GDGM93256	China	PX524002	PX704974	PX516740	Zhang et al. 2025
*C. parvoflavus*	Herrera204	Mexico	MT371338	MT449707	–	Montoya et al. 2021
*C. parvoflavus*	Herrera229	Mexico	MT371339	MT449708	–	Montoya et al. 2021
*C. parvoflavus*	Montoya5423 (HT)	Mexico	MT371337	MT449706	–	Montoya et al. 2021
*C. phloginus*	1690/V.A 13.152	Korea	–	KY271942	MW286816	Antonín et al. 2017; Buyck et al. 2021
***C. phloginus***	**FJAU81683**	**China**	**PZ379285**	**PZ424363**	**PZ435488**	**Present study**
***C. phloginus***	**FJAU81684**	**China**	**PZ379286**	**PZ426405**	**PZ435489**	**Present study**
*C. pseudoaustrosinensis*	GDGM53492	China	PX524018	PX704997	PX516758	Zhang et al. 2025
*C. pseudoaustrosinensis*	GDGM81426 (HT)	China	PX524021	PX704998	PX516760	Zhang et al. 2025
*C. romagnesianus*	PC0085043 (HT)	France	KX828806	–	–	Olariaga et al. 2017
*C. romagnesianus*	PC0142170	France	KX828808	–	–	Olariaga et al. 2017
*C. roseocastaneus*	GDGM91302 (HT)	China	PX523969	PX705001	PX516750	Zhang et al. 2025
*C. roseocastaneus*	GDGM91690	China	PX523966	PX705002	PX516751	Zhang et al. 2025
*C. sinocinnabarinus*	GDGM83230 (HT)	China	OM978984	ON119099	–	Zhang et al. 2022
*C. sinocinnabarinus*	GDGM83232	China	–	ON119100	ON119049	Zhang et al. 2022
C. aff. subcyanoxanthus	BB 00.1137 (HT)	Madagascar	–	JX192990	KF294734	Buyck et al. 2014
C. aff. subcyanoxanthus	BB 98. 014	Tanzania	KF294615	JX192973	KF294689	Buyck et al. 2014
C. cf. subcyanoxanthus	1609/EDC14.034	Zambia	KX857098	KX857072	KX857002	Buyck 2016
*C. subtilis*	GDGM81310 (HT)	China	PX524024	–	PX516761	Zhang et al. 2025
*C. tabernensis*	BB 07.056	USA	KF294631	GQ914974	KF294706	Buyck et al. 2011a, 2014
*C. tabernensis*	BB 07.119	USA	KF294634	GQ914976	KF294709	Buyck et al. 2011a, 2014
*C. texensis*	BB 07.120 (PT)	USA	JN940601	–	KF294710	Buyck et al. 2014
*C. texensis*	BB 07.018 (HT)	USA	KF294626	–	KF294701	Buyck et al. 2014
*C. umbonata*	317 (HT)	India	HM750922	–	–	Deepika et al. 2013
*C. xanthocyaneus*	1751/S.N. Ebika 2333	Republic of the Congo	MT006309	MT002277	–	Buyck et al. 2020b
*C. xanthocyaneus*	1752/S.N. Ebika 2353 (HT)	Republic of the Congo	MT006310	MT002278	–	Buyck et al. 2020b
*C. zangii*	GDGM83186	China	MZ605117	MZ614016	MZ614060	Zhang et al. 2021
*C. zangii*	GDGM83193	China	MZ605118	MZ614017	MZ614061	Zhang et al. 2021
*C. zangii*	HKAS 118077	China	–	ON089619	ON089628	Wang et al. 2023

The phylogenetic analysis was conducted according to the following workflow: (1) individual gene fragments (nrLSU, *tef1-α*, and *rpb2*) were independently aligned using MEGA7 (Kumar et al. 2016); (2) sequence alignments were manually adjusted using BioEdit v7.1.3 (Hall 1999); (3) concatenated alignments were generated using PhyloSuite 1.2.3 (Xiang et al. 2023); (4) the optimal evolutionary models for maximum likelihood (ML) and Bayesian inference (BI) analyses were determined using ModelFinder v2.2.0 based on the BIC and AICc criteria (Kalyaanamoorthy et al. 2017); (5) phylogenetic trees were inferred using IQ-TREE v1.6.8 (Nguyen et al. 2015) and MrBayes v3.2.6 (Ronquist et al. 2012). ML bootstrap support was assessed with 1,000 replicates. For Bayesian analysis, two parallel runs of 2,000,000 generations were performed, with the first 25% discarded as burn-in; convergence was achieved when the average standard deviation of split frequencies reached 0.004; (6) the resulting trees were visualized using the Interactive Tree of Life (iTOL) v7.5.1 (https://itol.embl.de) and annotated using Adobe Illustrator 2026 (Adobe, San Jose, CA, USA). The results of the phylogenetic analyses (BI and ML trees) and alignment data for the concatenated sequences were deposited in Figshare (https://doi.org/10.6084/m9.figshare.33001106).

## Results

### Molecular phylogeny

**Figure 1.**
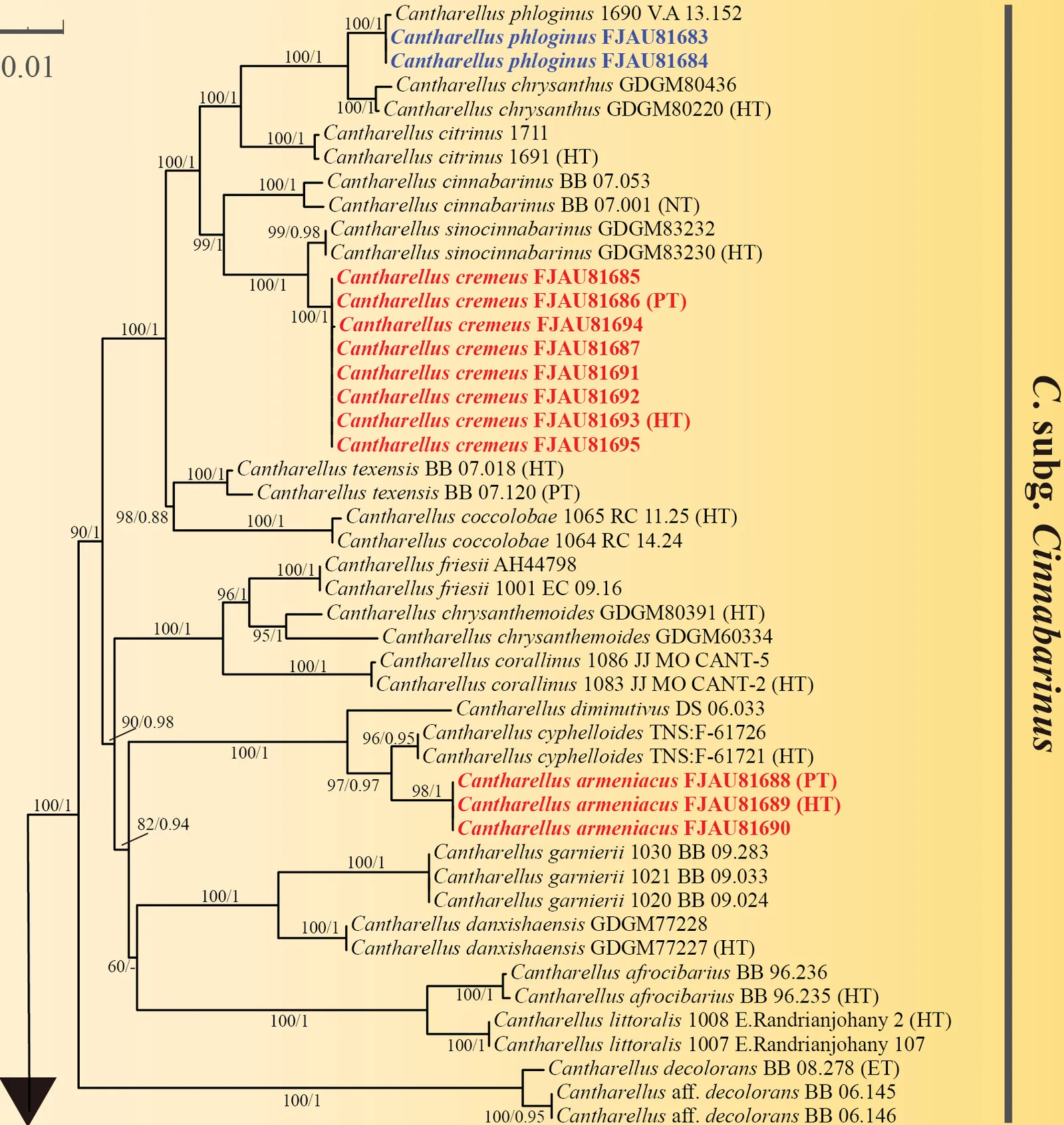
Phylogenetic tree of representative *Cantharellus* species based on the nrLSU–*tef1-α*–*rpb2* dataset inferred using ML and BI methods. Values at nodes indicate maximum likelihood bootstrap support (≥70%) and Bayesian posterior probabilities (≥0.90). Holotypes, epitypes, paratypes, and neotypes are indicated by (HT), (ET), (PT), and (NT), respectively. New taxa are shown in bold red, newly recorded taxa in bold purple, previously known taxa collected in this study in bold blue, and public database sequences in black.

**Figure 2.**
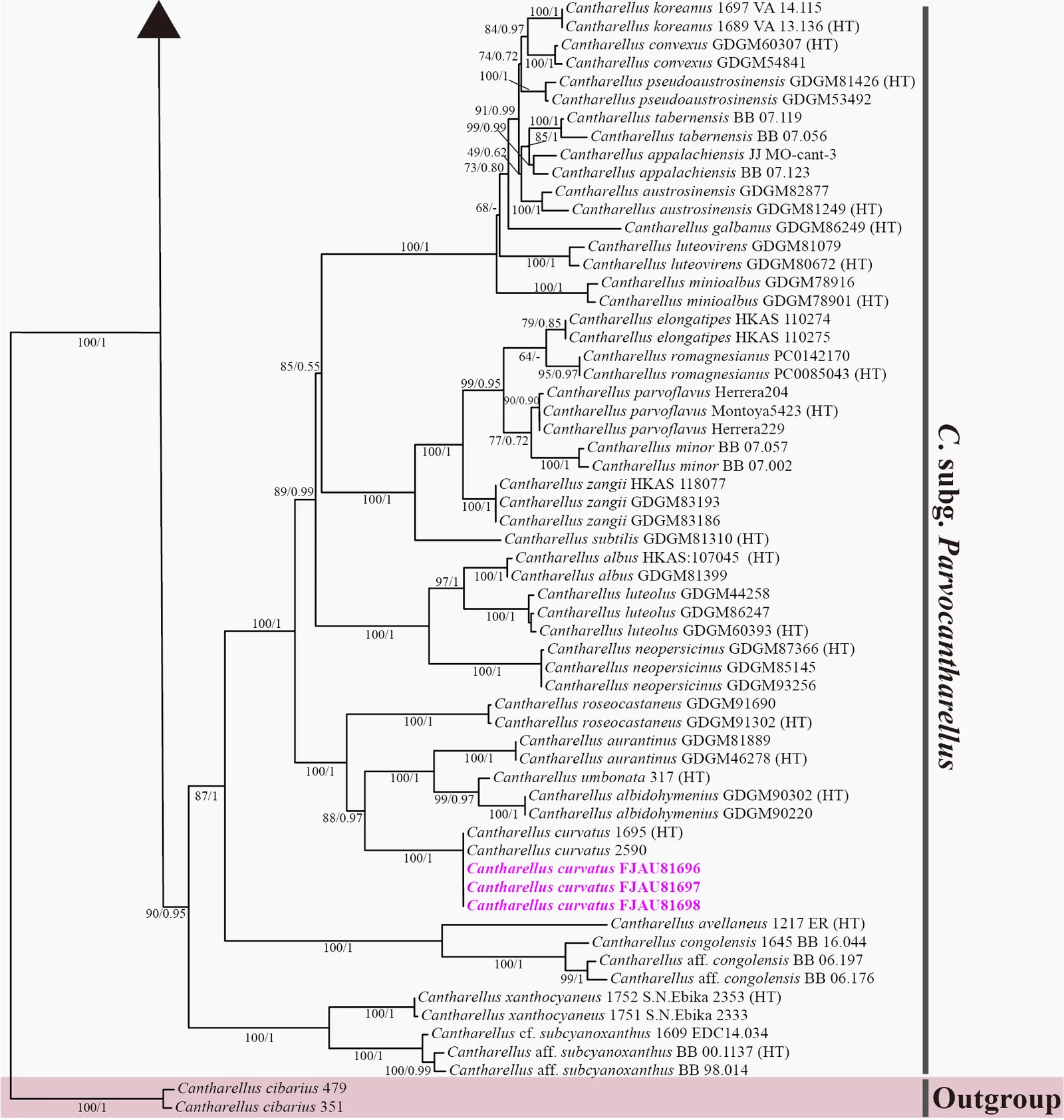
Phylogenetic tree of representative *Cantharellus* species based on the nrLSU–*tef1-α*–*rpb2* dataset inferred using ML and BI methods. Values at nodes indicate maximum likelihood bootstrap support (≥70%) and Bayesian posterior probabilities (≥0.90). Holotypes, epitypes, paratypes, and neotypes are indicated by (HT), (ET), (PT), and (NT), respectively. New taxa are shown in bold red, newly recorded taxa in bold purple, previously known taxa collected in this study in bold blue, and public database sequences in black.

In the phylogenetic analyses conducted for this study, 48 sequences were newly generated, comprising 16 sequences each for nrLSU, *tef1-α*, and *rpb2*. The concatenated dataset included a total of 275 sequences derived from 108 fungal collections, including 94 for nrLSU, 95 for *tef1-α*, and 86 for *rpb2*. The alignment, including gaps, encompassed 2,785 characters, with positions 1–1,073 corresponding to nrLSU, 1,074–1,857 to *tef1-α*, and 1,858–2,785 to *rpb2*. In the concatenated alignment, 1,848 (66.35%) of the characters were constant, 104 were singletons, 833 (29.91%) were parsimony-informative, and 1,156 distinct patterns were identified. For ML, the best-fit models were TIM3+F+R3 (nrLSU), TIMe+I+G4 (*tef1-α*), and TIM3e+G4 (*rpb2*); for BI, the models were K2P+I+G4 (nrLSU), K2P+I+G4 (*tef1-α*), and K2P+G4 (*rpb2*). ML and BI analyses yielded nearly identical topologies without significant topological conflicts; therefore, only the ML tree was presented (Figs 1, 2). Based on newly collected specimens from the Dabie Mountains, China, three highly supported clades within subg. *Cinnabarinus* were identified, comprising two novel species and one previously documented Chinese species. Furthermore, one species belonging to subg. *Parvocantharellus* was reported as a new record for Anhui Province, China.

### Taxonomy

#### Cantharellus cremeus

Taxon classificationFungiScleractiniaFungiidae

H. Chu, Y.L. Tuo & B. Zhang sp. nov.

7BEAB9AA-8D0B-5835-90E8-B8207B166EE6

863695

Figs 3A–E, 7A–C

##### Etymology.

The specific epithet “cremeus” refers to the creamy-white to pale yellow coloration of the basidiomata.

**Figure 3.**
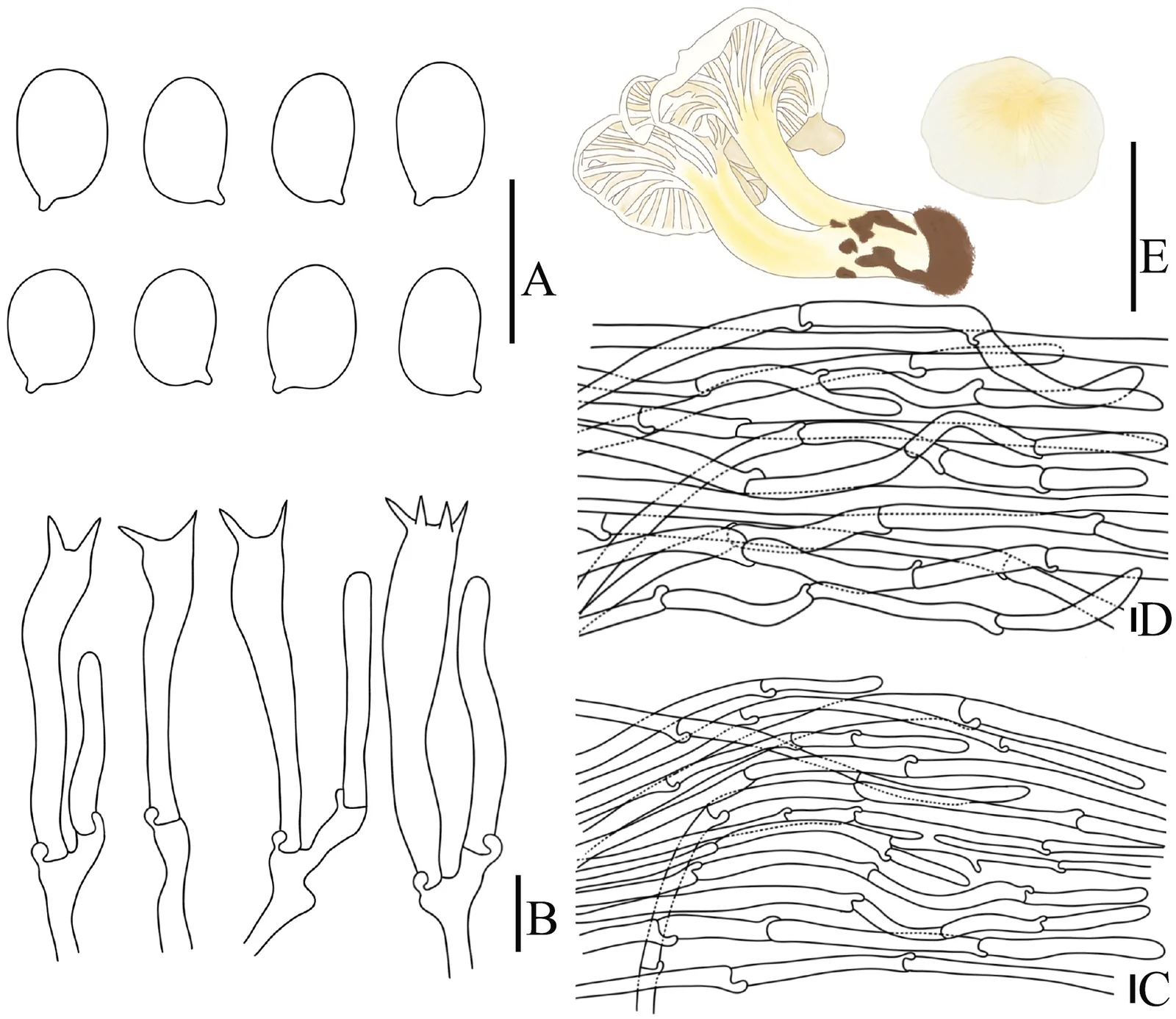
Macroscopic and microscopic features of *Cantharellus cremeus* (FJAU81693, holotype). **A**. Basidiospores; **B**. Basidia; **C**. Stipitipellis; **D**. Pileipellis; **E**. Basidiomata. Scale bars: 10 μm (**A–D**); 10 mm (**E**).

##### Holotype.

China, • Anhui Province, Lu’an City, Huoshan County, Baimajian Peak Scenic Area, 31°08'15.41"N, 116°11'30.82"E, 22 August 2024, Y.L. Tuo (FJAU81693). GenBank: nrLSU = PZ379279, *tef*1-α = PZ426413, *rpb*2 = PZ435498.

##### Paratype.

China, • Anhui Province, Lu’an City, Huoshan County, Baimajian Peak Scenic Area, 31°08'15.41"N, 116°11'30.82"E, 9 August 2023, Y.L. Tuo (FJAU81686). GenBank: nrLSU = PZ379272, *tef*1-α = PZ426407, *rpb*2 = PZ435491.

##### Diagnosis.

Distinguished by a white to pale yellow pileus, a well-developed white to pale yellow hymenophore, a pale yellow to bright yellow stipe, and broadly ellipsoid to ellipsoid basidiospores.

##### Description.

Basidiomata very small to small. Pileus 4–15 mm wide, applanate, centrally depressed, imperforate; margin slightly incurved when young, becoming straight to slightly recurved at maturity, surface smooth; marginal white (1A1), central portion usually darker, mostly white to pastel yellow (3A1–3A4) or greyish yellow to olive yellow (3C3–3C6), no staining. Context white, thin, no staining. Hymenophore decurrent, well-developed; distant, with or without transverse veins, occasionally forked near the pileus margin, white to pastel yellow (3A1–3A4), concolourous with the pileus, no staining. Stipe 7–13 × 0.5–1 mm, cylindrical, slightly tapered towards the base, glabrous, solid, pastel yellow to vivid yellow (3A4–3A8), no staining. Odor indistinct.

Basidiospores 6.4–8.2 × 4.1–5.8 μm, *Lm* × *Wm* = 7.19 (± 0.43) × 4.94 (± 0.36) μm, *Q* = (1.27–) 1.32–1.67 (–1.68), *Qm* = 1.46 ± 0.10, broadly ellipsoid to elongate ellipsoid, smooth, thin-walled, hyaline to yellowish in 5% KOH, with oil droplets and granular inclusions, inamyloid. Basidia 42–65 × 7–10 μm, clavate, with 2–6 sterigmata; sterigmata 4–7 μm long, hyaline to yellowish in 5% KOH, with granular contents. Basidioles abundant, clavate, hyaline to yellowish in 5% KOH, with granular contents. Hymenophoral trama composed of cylindrical, thin-walled, branched hyphae, hyaline to yellowish in 5% KOH, 3–12 μm wide. Subhymenium filamentous. Cystidia absent. Pileipellis composed of cylindrical, thin-walled, branched hyphae, hyaline in 5% KOH, 4–13 μm wide; terminal cells (40–) 42–80 (–83) × 6–10 (–11) μm, cylindrical, thin-walled, sometimes slightly curved, slightly constricted near apex. Stipitipellis composed of cylindrical, thin-walled, branched hyphae, hyaline in 5% KOH, 3–9 μm wide. Clamp connections distinct and abundant in all tissues.

##### Habitat and distribution.

Gregarious to scattered within mixed forests dominated by *Quercus serrata* and other broad-leaved trees. The specimen was collected approximately 1.6 m away from a *Pinus* sp. with a diameter at breast height (DBH) of 27.3 cm. Currently, this species is only known from the Dabie Mountains in Anhui Province, China.

##### Additional specimens examined.

China, • Anhui Province, Lu’an City, Huoshan County, Baimajian Peak Scenic Area, 31°08'15.41"N, 116°11'30.82"E, 9 August 2023, Y.L. Tuo (FJAU81685), GenBank: nrLSU = PZ379271, *tef*1-α = PZ426406, *rpb*2 = PZ435490; • Same location, in a *Rhododendron* L. forest, 9 August 2023, Y.L. Tuo (FJAU81687), GenBank: nrLSU = PZ379273, *tef*1-α = PZ426408, *rpb*2 = PZ435492; • Same location, 3 August 2024, Y.L. Tuo (FJAU81691), GenBank: nrLSU = PZ379277, *tef*1-α = PZ426410, *rpb*2 = PZ435496; • Same location, 3 August 2024, Y.L. Tuo (FJAU81692), GenBank: nrLSU = PZ379278, *tef*1-α = PZ426411, *rpb*2 = PZ435497; • Same location, 3 August 2024, Y.L. Tuo (FJAU81694), GenBank: nrLSU = PZ379280, *tef*1-α = PZ426414, *rpb*2 = PZ435499; • Same location, in a mixed forest of *Quercus serrata* Thunb. and *Rhododendron* L., 3 August 2024, Y.L. Tuo (FJAU81695), GenBank: nrLSU = PZ379281, *tef*1-α = PZ426415, *rpb*2 = PZ435500.

##### Notes.

This species is classified within *Cantharellus* subg. *Cinnabarinus* and is distinguished by a white to pale yellow pileus, a well-developed pale yellow lamellate hymenophore, and a pale yellow stipe, in addition to basidia 42–65 × 7–10 μm and basidiospores measuring 6.4–8.2 × 4.1–5.8 μm.

Morphologically, *Cantharellus cremeus* resembles *Cantharellus cyphelloides* Suhara & S. Kurogi, which belongs to the same subgenus, as both form small, whitish basidiomata. However, *C. cremeus* can be distinguished from *C. cyphelloides* by its smaller pileus (4–15 mm vs. up to 20 mm diam.), lamellate (vs. smooth) hymenophore, indistinct (vs. strong) odor, and smaller basidiospores (6.4–8.2 × 4.1–5.8 μm vs. 7.5–10 × 4.5–7 μm) (Suhara and Kurogi 2015). Additionally, although it shares whitish basidiomata with *Cantharellus albus* S.P. Jian & B. Feng, which belongs to subg. *Parvocantharellus*, it differs in having a smaller pileus (4–15 mm vs. 20–50 mm diam.), a shorter stipe (7–13 × 0.5–1 mm vs. 20–70 × 2–10 mm), basidiospores with a higher *Q* value (6.4–8.2 × 4.1–5.8 μm, *Q* = 1.32–1.67 vs. 6–8 × 5–7 μm, *Q* = 1–1.4), and the absence (vs. presence) of yellowish discoloration upon bruising (Jian et al. 2020).

Phylogenetically, *Cantharellus cremeus* is sister to *Cantharellus sinocinnabarinus* Ming Zhang, S.C. Shao & T.H. Li, but differs in possessing a white to pale yellow (vs. orange-red) pileus, a solid (vs. hollow) stipe, smaller basidiospores (6.4–8.2 × 4.1–5.8 μm vs. 7–9 × 5–6 μm), and shorter basidia (42–65 × 7–10 μm vs. 50–75 × 10–12 μm) (Zhang et al. 2022). These consistent morphological distinctions, together with its phylogenetic position, support its recognition as a distinct species.

#### Cantharellus armeniacus

Taxon classificationFungiScleractiniaFungiidae

H. Chu, Y.L. Tuo & B. Zhang sp. nov.

D7E5E107-3946-5D99-B719-14CC4A54996D

863696

Figs 4A–E, 7D–F

##### Etymology.

The specific epithet “armeniacus” refers to the bright yellow to vivid yellow coloration of the basidiomata, resembling that of the flesh of an apricot.

**Figure 4.**
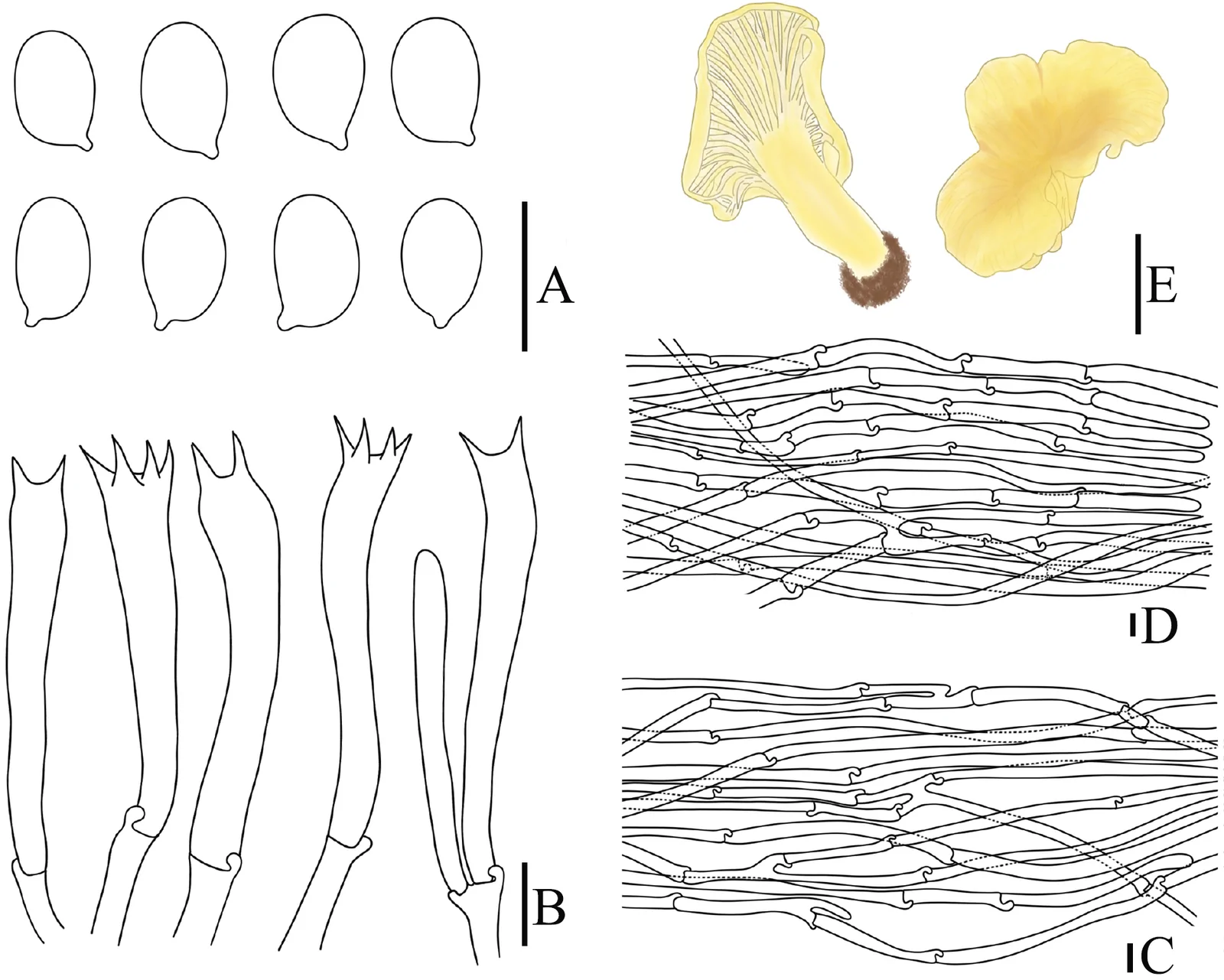
Macroscopic and microscopic features of *Cantharellus armeniacus* (FJAU81689, holotype). **A**. Basidiospores; **B**. Basidia; **C**. Stipitipellis; **D**. Pileipellis; **E**. Basidiomata. Scale bars: 10 μm (**A–D**); 10 mm (**E**).

##### Holotype.

China, • Anhui Province, Lu’an City, Jinzhai County, Yanzihe Town, 31°17'05.94"N, 115°56'50.15"E, 3 July 2024, Y.L. Tuo (FJAU81689), GenBank: nrLSU = PZ379275, *tef*1-α = PZ426409, *rpb*2 = PZ435494.

##### Paratype.

China, • Anhui Province, Lu’an City, Jinzhai County, Yanzihe Town, 31°17'05.94"N, 115°56'50.15"E, 12 October 2023, Y.L. Tuo (FJAU81688), GenBank: nrLSU = PZ379274, *tef*1-α = PZ435484, *rpb*2 = PZ435493.

##### Diagnosis.

Distinguished by its small, bright yellow to vivid yellow basidiomata, a usually finely tomentose pileus, a well-developed lamellate hymenophore, ellipsoid basidiospores, small basidia, and thin-walled hyphae of the pileipellis.

##### Description.

Basidiomata small. Pileus 12–36 mm wide, convex when young with margin slightly incurved, becoming applanate with margin slightly curved to nearly straight at maturity, centrally depressed and infundibuliform when aged, occasionally perforate; margin undulate and faintly lobed; surface glabrous or tomentose, no staining; greyish yellow to orange yellow (4B5–4B7) when young, light yellow to vivid yellow (3A5–3A8) at maturity. Context fleshy, yellow, no staining. Hymenophore decurrent, well-developed; folds less than 1.5 mm high, distant, occasionally forking near the pileus margin, with anastomosing veins between folds, light yellow to vivid yellow (3A5–3A8), concolourous with the pileus, no staining. Stipe 10–28 × 2–10 mm, fleshy, central, cylindrical or slightly tapered downward, finely tomentose, solid, becoming hollow upon drying, light yellow to vivid yellow (3A5–3A8), concolourous with the pileus, no staining. Odor mild, with an apricot-like fragrance.

Basidiospores 6.1–8.1 × 4.1–5.7 μm, *Lm* × *Wm* = 7.15 (± 0.50) × 4.81 (± 0.37) μm, *Q* = (1.29–) 1.33–1.65 (–1.71), *Qm* = 1.49 ± 0.10, broadly ellipsoid to elongate ellipsoid, smooth, thin-walled, with contents, hyaline to pale yellow in 5% KOH, inamyloid. Basidia subclavate, 35–72 × 5–9 μm, with 1–5 (mostly 2–4) sterigmata, sterigmata 1–8 μm long, some extremely short, hyaline to pale yellow in 5% KOH, with granular contents. Basidioles abundant, clavate, smooth, thin-walled, hyaline to pale yellow in 5% KOH, with granular contents. Hymenophoral trama composed of cylindrical, thin-walled, branched hyphae, hyaline to pale yellow in 5% KOH, some with contents, 3–7 μm wide. Subhymenium filamentous. Cystidia absent. Pileipellis hyphae cylindrical or clavate, thin-walled, some irregularly inflated and branched, hyaline to pale yellow in 5% KOH, 2–14 μm wide; terminal cells (40–) 47–87 (–94) × 5–8 (–11) μm, clavate, thin-walled, some with contents, slightly constricted near apex, hyaline to pale yellow in 5% KOH. Stipitipellis hyphae cylindrical, thin-walled, branched, occasionally irregularly inflated, hyaline to pale yellow in 5% KOH, 2–10 μm wide. Clamp connections distinct and abundant in all tissues.

##### Habitat and distribution.

Scattered in mixed forests of *Quercus aliena* Bl. and other broad-leaved trees. The specimen was collected approximately 1.5 m away from a *Quercus aliena* (DBH: 18.5 cm). Currently, it is only known from the Dabie Mountains, Anhui Province, China.

##### Additional specimens examined.

China, • Anhui Province, Lu’an City, Jinzhai County, Yanzihe Town, 31°17'05.94"N, 115°56'50.15"E, 14 July 2024, Y.L. Tuo (FJAU81690). GenBank: nrLSU = PZ379276, *tef*1-α = PZ435485, *rpb*2 = PZ435495.

##### Notes.

This species belongs to *Cantharellus* subg. *Cinnabarinus* and is characterized by small, entirely bright yellow basidiomata, a well-developed lamellate hymenophore with bifurcate veins, a solid stipe, relatively small basidiospores, and slender basidia.

Morphologically, *Cantharellus armeniacus* shares yellow basidiomata with several members of *Cantharellus* subg. *Cinnabarinus*. It can be distinguished from its relatives by a combination of morphological characters. It differs from *C. diminutivus* Corner in having a finely tomentose, non-squamulose (vs. squamulose) pileus, a solid (vs. hollow) stipe, and smaller basidiospores (6.1–8.1 × 4.1–5.7 μm vs. 7–9 × 4.75–6 μm) (Eyssartier et al. 2008). In contrast to *C. fistulosus* Tibuhwa & Buyck, it has a bright yellow (vs. pink) hymenophore, a solid (vs. hollow) stipe, larger basidiospores (6.1–8.1 × 4.1–5.7 μm vs. 5–7 × 3.5–4 μm), and larger pileipellis terminal cells (40–94 × 5–11 μm vs. 13–65 × 3–5 μm) (Tibuhwa et al. 2008). It can be separated from *C. chrysanthus* Ming Zhang, C.Q. Wang & T.H. Li by its bright yellow (vs. pinkish white) hymenophore, smaller basidiospores (6.1–8.1 × 4.1–5.7 μm vs. 7.5–9 × 5–6.5 μm), and shorter basidia (35–72 × 5–9 μm vs. 55–100 × 7–11 μm) (Zhang et al. 2022). It differs from *C. citrinus* Buyck, R. Ryoo & Antonín, including the material reported from Guizhou, China, in having a solid (vs. hollow) and finely tomentose (vs. glabrous) stipe (Buyck et al. 2020a; Zhang et al. 2022).

In addition, it can be distinguished from four other yellow-colored species of this subgenus. It differs from the tropical African *C. afrocibarius* Buyck & V. Hofst. in having a *Fagaceae* (vs. *Fabaceae*) host (Buyck et al. 2013). In contrast to *C. danxiashanensis* Ming Zhang & T.H. Li, it has a broader stipe (10–28 × 2–10 mm vs. 7–20 × 1–2 mm) (Zhang et al. 2025). It can be separated from *C. garnierii* Ducousso & Eyssart. by its smaller basidiospores (6.1–8.1 × 4.1–5.7 μm vs. 8–9.25 × 5.3–6 μm) (Ducousso et al. 2004). It differs from *C. chrysanthemoides* Ming Zhang in having basidiospores with a different *Q* value (6.1–8.1 × 4.1–5.7 μm, *Q* = 1.33–1.65, *Qm* = 1.49 vs. 7–8 × 5–6 μm, *Q* = 1.27–1.4, *Qm* = 1.33) (Zhang et al. 2025).

Phylogenetically, this species is sister to *C. cyphelloides* but differs markedly in both macromorphological and micromorphological traits. Macromorphologically, it is distinguished from *C. cyphelloides* by its finely tomentose (vs. glabrous) pileus, lamellate and bright yellow (vs. smooth and cream-yellow) hymenophore, and distinctly longer and wider stipe (10–28 × 2–10 mm vs. 2–5 × 1–2 mm). Micromorphologically, it has smaller basidiospores (6.1–8.1 × 4.1–5.7 μm vs. 7.5–10 × 4.5–7 μm) and slightly narrower basidia (35–72 × 5–9 μm vs. 50–74 × 7–11.5 μm) (Suhara and Kurogi 2015). These consistent morphological distinctions, together with its phylogenetic position, support its recognition as a distinct species.

#### Cantharellus phloginus

Taxon classificationFungiScleractiniaFungiidae

S.C. Shao & P.G. Liu, Mycoscience 57(2): 146 (2016)

0E548E4C-D538-546D-9F3B-2FE8CADAB4FE

Figs 5A–E, 7G–I

= Cantharellus albovenosus Buyck, Antonín & Ryoo, Mycological Progress 16(8): 757 (2017).

##### Description.

Basidiomata small. Pileus 8–40 mm wide, convex with slightly incurved margin when young, becoming applanate with slightly curved margin at maturity, centrally depressed and infundibuliform when aged, non-perforate; margin undulate and faintly lobed; surface tomentose when young, then glabrous, no staining, vivid orange red to pastel red (8A5–8A8) or deep orange to pale orange (6A3–6A8) from the center to margin, margin slightly paler, pale yellow (1A3) with reddish tinge when dried. Context fleshy and white, pale yellow (1A3), pale red (7A3) or pale orange (6A3) beneath pileipellis, no staining. Hymenophore decurrent, well-developed; folds 0.5–2 mm high, moderately distant, forking towards the pileus margin, with anastomosing veins between folds that are more distinct at margin; white to pale yellow (1A1–1A3), no staining. Stipe 14–43 × 3.5–12 mm, fleshy, central, cylindrical or slightly enlarged at base, tomentose, solid, concolourous with or slightly paler than pileus, pale red to pastel red (8A3–8A5) or pale orange to pale yellow (1A3–6A3), no staining. Odor mild, with a strong apricot-like fragrance.

**Figure 5.**
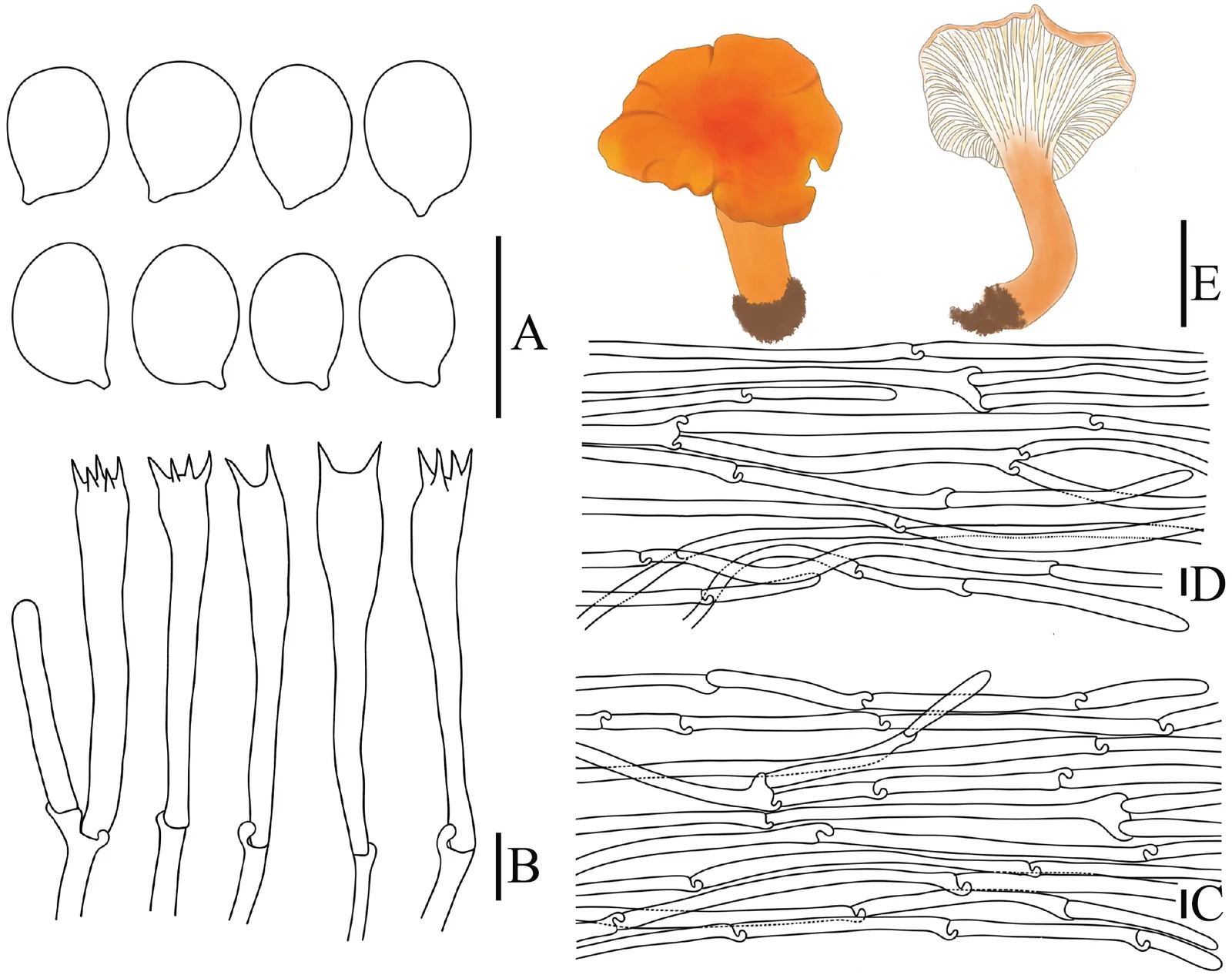
Macroscopic and microscopic features of *Cantharellus phloginus* (FJAU81683). **A**. Basidiospores; **B**. Basidia; **C**. Stipitipellis; **D**. Pileipellis; **E**. Basidiomata. Scale bars: 10 μm (**A–D**); 10 mm (**E**).

Basidiospores 6.8–8.6 × 4.8–6.2 μm, *Lm* × *Wm* = 7.59 (± 0.55) × 5.32 (± 0.37) μm, *Q* = (1.24–) 1.29–1.60 (–1.65), *Qm* = 1.43 ± 0.10, broadly ellipsoid to elongate ellipsoid, occasionally ellipsoid, smooth, thin-walled, with contents, pale yellow in 5% KOH, inamyloid. Basidia clavate, 46–85 × 7–12 μm, with 2–5 sterigmata, sterigmata 4–9 μm long, hyaline to pale yellow in 5% KOH, with contents. Basidioles abundant, clavate to subcylindrical, smooth, with contents, hyaline to pale yellow in 5% KOH. Hymenophoral trama composed of subcylindrical, thin-walled, branched hyphae, with contents, hyaline to pale yellow in 5% KOH, 2–9 μm wide. Subhymenium filamentous. Cystidia absent. Pileipellis composed of parallel or interwoven, cylindrical, thin-walled, branched hyphae, some with contents, hyaline to pale yellow in 5% KOH, 2–13 μm wide; terminal cells (38–) 45–98 (–103) × (4–) 5–8 (–9) μm, subcylindrical or clavate, obtuse or slightly constricted at apex, thin-walled, hyaline to pale yellow in 5% KOH, with contents. Stipitipellis composed of subcylindrical, thin-walled, branched hyphae, some with contents, hyaline to pale yellow in 5% KOH, 2–11 μm wide. Clamp connections distinct and abundant in all tissues.

##### Habitat and distribution.

Scattered to gregarious in mixed forests dominated by *Quercus variabilis* Bl. and other broad-leaved trees. The specimen was collected approximately 0.1 m away from the base of a *Quercus variabilis* tree (DBH: 10.6 cm). This species is currently known from Anhui, Hebei, Hubei, Jiangsu, Shandong, Yunnan, and Zhejiang provinces of China, as well as from the Republic of Korea (Huang et al. 2026).

##### Additional specimens examined.

China, • Anhui Province, Lu’an City, Jinzhai County, Tiantangzhai Town, 31°12'50.52"N, 115°48'22.58"E, 18 July 2023, Y.L. Tuo (FJAU81683). GenBank: nrLSU = PZ379285, *tef*1-α = PZ424363, *rpb*2 = PZ435488; • Same location, 18 July 2023, Y.L. Tuo (FJAU81684). GenBank: nrLSU = PZ379286, *tef*1-α = PZ426405, *rpb*2 = PZ435489.

##### Notes.

This species belongs to *Cantharellus* subg. *Cinnabarinus* and is characterized by its vivid red or yellow basidiomata, a well-developed white to pale yellow lamellate hymenophore, a stipe concolorous with or slightly paler than the pileus, and ellipsoid basidiospores measuring 7.5–8.5 × 5–6.5 μm (Shao et al. 2016).

The concept of species delimitation has been comprehensively developed in recent decades. *Cantharellus phloginus* was originally described by Shao et al. (2016) based on specimens collected from southwestern China. Subsequently, Antonín et al. (2017) described *C. albovenosus* from the Republic of Korea, noting significant morphological differences and divergence in a single-marker phylogenetic analysis compared with *C. phloginus*. However, Wang et al. (2023) demonstrated that the genetic divergence between these two taxa was not supported in a multi-marker phylogeny based on ITS, nrLSU, *tef1-α*, and *rpb2* sequences, leading them to propose *C. albovenosus* as a synonym of *C. phloginus*. Initially, Zhang et al. (2022) maintained these as distinct species; however, Zhang et al. (2025) subsequently aligned with the taxonomic interpretation of Wang et al. (2023).

The present species agrees well with the protologue of *C. phloginus* in both macroscopic and micromorphological characters, with basidiospores measuring 6.8–8.6 × 4.8–6.2 μm (vs. 7.5–8.5 × 5–6.5 μm) (Shao et al. 2016). It also agrees well with the protologue of *C. albovenosus* (first reported from Korea and later from China), except for the pileus color: the pileus is orange-red, whereas that of *C. albovenosus* is yellow; the basidiospores measure 6.8–8.6 × 4.8–6.2 μm in the present study, 7.2–9.0 × 5.0–6.0 μm in the type from Korea, and 7.0–8.5 × 5.0–6.0 μm in the Chinese material (Buyck et al. 2020a; Zhang et al. 2022). Given that basidiomatal albinism has been documented in several *Cantharellus* species (Olariaga et al. 2015), and in light of combined morphological and phylogenetic evidence, the present study supports the treatment of *C. albovenosus* as a synonym of *C. phloginus*.

#### Cantharellus curvatus

Taxon classificationFungiScleractiniaFungiidae

Buyck, R. Ryoo & Antonín, MycoKeys 76: 39 (2020)

96FF75DD-5BDB-5CBD-B311-2D84128B2844

Figs 6A–E, 7J–L

##### Description.

Basidiomata very small to small. Pileus 9–38 mm wide, convex with slightly incurved margin when young, becoming applanate with margin slightly curved to nearly straight at maturity, centrally depressed and infundibuliform when aged, non-perforate; margin undulate and faintly lobed; surface glabrous, no staining, greyish yellow to orange yellow (4B5–4B7) when young, light yellow to vivid yellow (3A5–3A8) at maturity. Context fleshy, pale yellow (1A3), thin, no staining. Hymenophore decurrent and well-developed; folds less than 1.5 mm high, distant, occasionally forking near the pileus margin, with weakly anastomosing veins between the folds near the margin; white to pale yellow (1A1–1A3), no staining. Stipe 7–29 × 1.5–5 mm, fleshy, central, cylindrical or slightly widened downward, glabrous, solid, white (1A1) initially, becoming light yellow to vivid yellow (3A5–3A8) at maturity, concolourous with the pileus, no staining. Odor mild, with a faint apricot-like fragrance.

**Figure 6.**
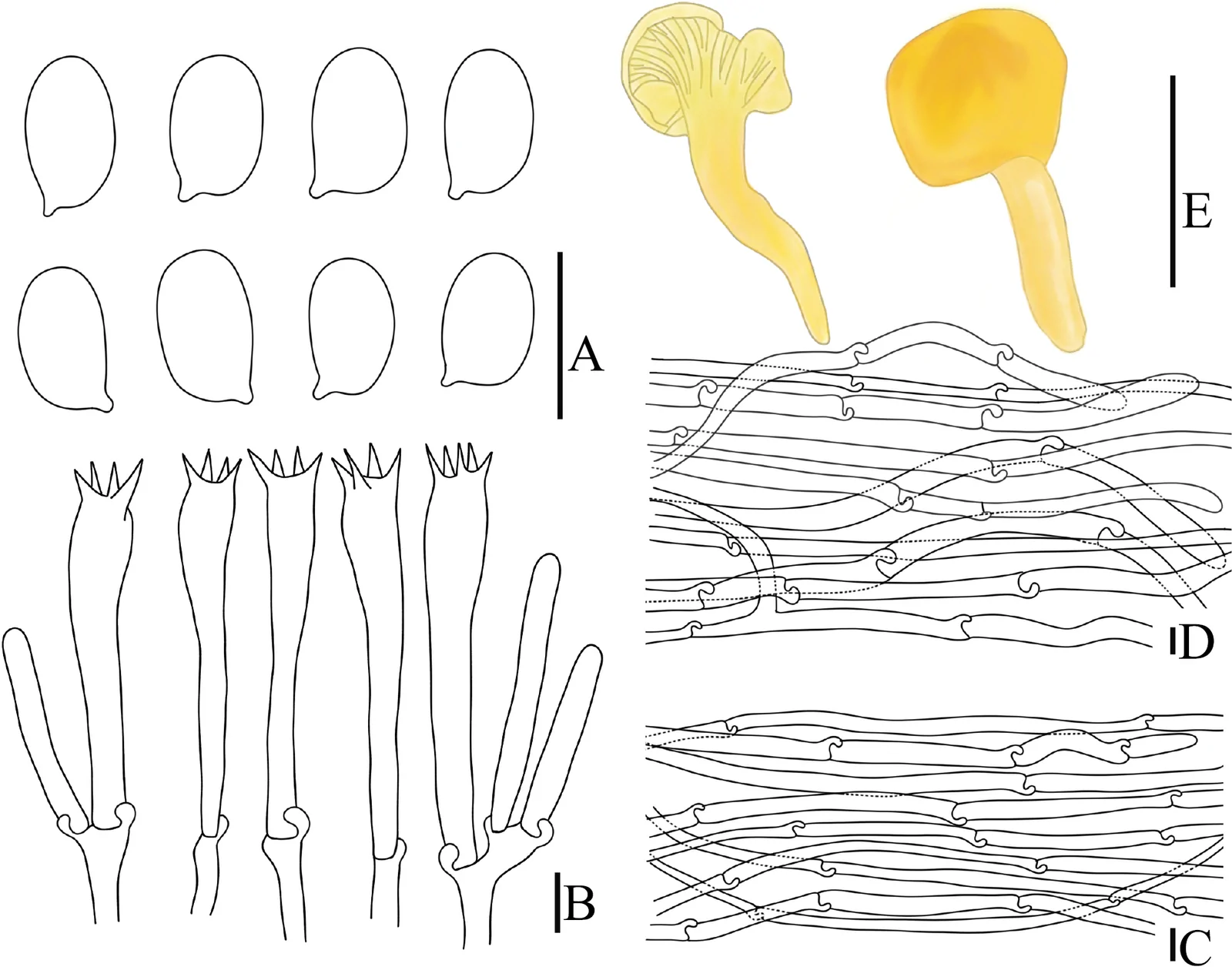
Macroscopic and microscopic features of *Cantharellus curvatus* (FJAU81696). **A**. Basidiospores; **B**. Basidia; **C**. Stipitipellis; **D**. Pileipellis; **E**. Basidiomata. Scale bars: 10 μm (**A–D**); 10 mm (**E**).

**Figure 7.**
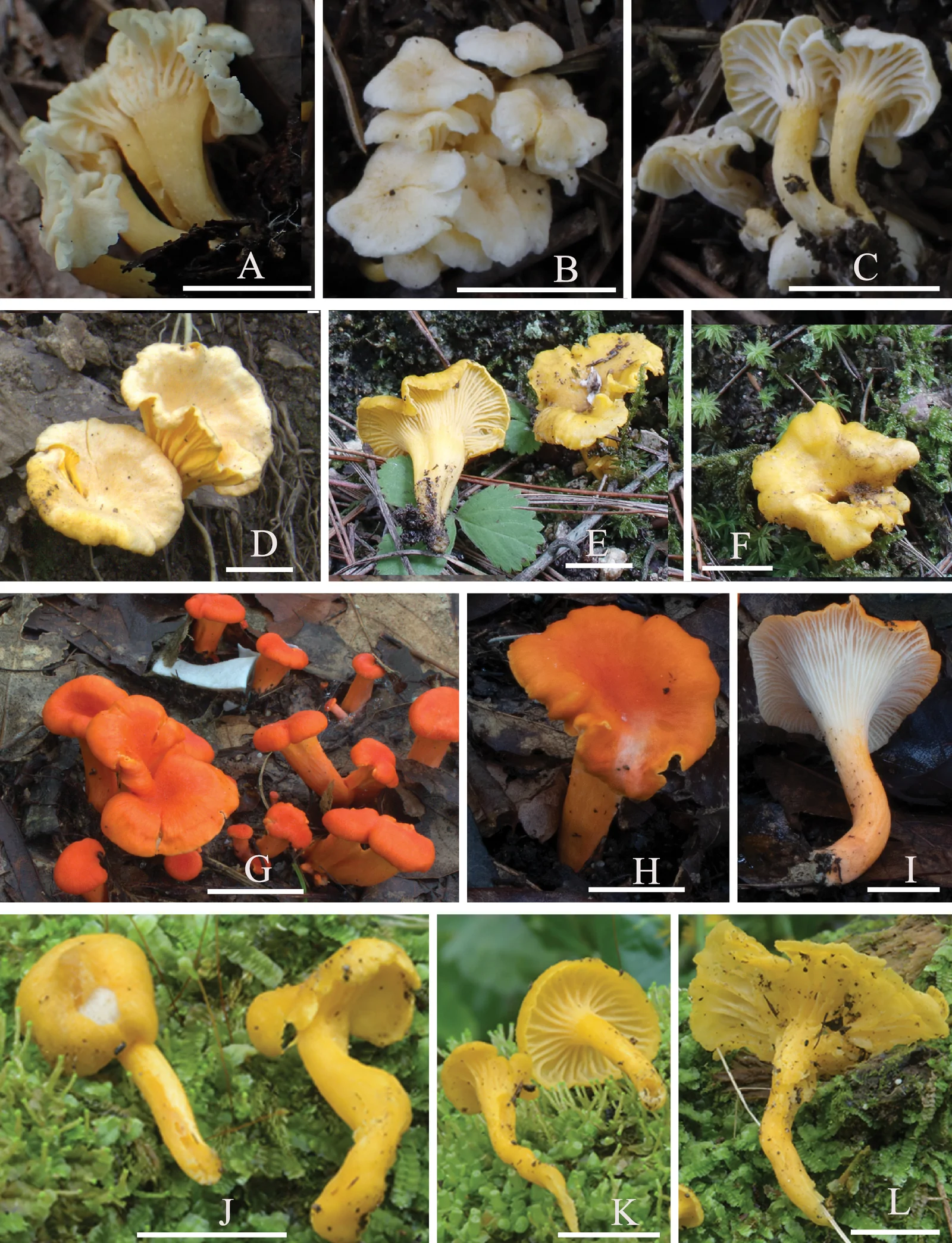
Species of *Cantharellus* from the Dabie Mountains. **A–C**. *C. cremeus*: **A**. FJAU81686, paratype; **B, C**. FJAU81693, holotype. **D–F**. *C. armeniacus*: **D**. FJAU81688, paratype; **E, F**. FJAU81689, holotype. **G–I**. *C. phloginus*: **G**. FJAU81683; **H, I**. FJAU81684. **J–L**. *C. curvatus* (FJAU81696). Scale bars: 10 mm (**A–L**).

Basidiospores 7.3–9.3 × 4.3–5.8 μm, *Lm* × *Wm* = 8.12 (± 0.45) × 4.89 (± 0.32) μm, *Q* = (1.47–) 1.51–1.78 (–1.85), *Qm* = 1.65 ± 0.09, ellipsoid to elongate ellipsoid, smooth, thin-walled, colorless to pale yellow in 5% KOH, inamyloid. Basidia clavate, 43–77 × 7–11 μm, with 4–6 sterigmata, sterigmata 4–7 μm long, colorless to pale yellow in 5% KOH, with contents. Basidioles abundant, subclavate, colorless to pale yellow in 5% KOH, with contents. Hymenophoral trama composed of cylindrical, thin-walled, branched hyphae, colorless in 5% KOH, 2–14 μm wide. Subhymenium filamentous. Cystidia absent. Pileipellis composed of parallel or interwoven, cylindrical, thin-walled, occasionally branched hyphae, hyaline to pale yellow in 5% KOH, 3–12 μm wide; terminal cells (56–) 62–93 (–98) × 6–9 (–10) μm, cylindrical, clavate or tapering toward the apex, thin-walled, hyaline to pale yellow in 5% KOH, with contents. Stipitipellis composed of cylindrical, thin-walled, occasionally branched hyphae, hyaline to pale yellow in 5% KOH, 3–12 μm wide. Clamp connections distinct and abundant in all tissues.

##### Habitat and distribution.

Scattered in mixed forests dominated by *Castanea mollissima* Bl. and other tree species. The specimen was collected approximately 2.6 m away from a *Pinus* sp. (DBH: 32.2 cm). This species was first reported from the Republic of Korea (Buyck et al. 2020a) and has more recently been documented from Heilongjiang and Shandong provinces in China (Wang et al. 2023).

##### Additional specimens examined.

China, • Anhui Province, Lu’an City, Jinzhai County, Tianma National Nature Reserve, 31°09'41.41"N, 116°25'56.45"E, 14 August 2024, Y.L. Tuo (FJAU81696). GenBank: nrLSU = PZ379282, *tef*1-α = PZ435486, *rpb*2 = PZ435501; China, • Anhui Province, Lu’an City, Jinzhai County, Tiantangzhai Town, 31°12'13.59"N, 115°48'22.05"E, 12 September 2024, Y.L. Tuo (FJAU81697). GenBank: nrLSU = PZ379283, *tef*1-α = PZ426412, *rpb*2 = PZ435502; • Same location, 28 July 2024, H. Chu (FJAU81698). GenBank: nrLSU = PZ379284, *tef*1-α = PZ435487, *rpb*2 = PZ435503.

##### Notes.

This species was originally described from the Republic of Korea and belongs to *Cantharellus* subg. *Parvocantharellus*. It is characterized by small, bright yellow to vivid yellow basidiomata, a well-developed white to pale yellow lamellate hymenophore with weakly anastomosing veins between folds near the margin, ellipsoid basidiospores, and thin-walled terminal cells in the pileipellis (Buyck et al. 2020a). Compared with the holotype, the present species differs in having slightly longer basidia (43–77 × 7–11 μm vs. 42–55 × 9.5–12 μm) and less distinctly curved (vs. distinctly curved) pseudocystidia. Prior to this study, the species was known in China only from Heilongjiang and Shandong provinces based on molecular data (Wang et al. 2023). This constitutes a new provincial record for Anhui Province.

### Key to *Cantharellus* species reported from the Dabie Mountains region

**Table:** 

1	Hymenophore smooth to nearly so	**2**
–	Hymenophore with well-defined gill-like folds	**4**
2	Stipe hollow	***C. hainanensis***
–	Stipe solid	**3**
3	Basidiospores 9–11 × 6.8–7.5 μm	***C. magnus***
–	Basidiospores 7–9 × 5–6 μm	***C. pseudohainanensis***
4	Stipe hollow	***C. elongatipes***
–	Stipe solid	**5**
5	Basidiomata white to nearly so	***C. cremeus***
–	Basidiomata not white	**6**
6	Basidiomata red	***C. phloginus***
–	Basidiomata yellow	**7**
7	Pileipellis with thick-walled hyphae	**8**
–	Pileipellis lacking thick-walled hyphae	**9**
8	Pileus surface squamulose	***C. vaginatus***
–	Pileus surface glabrous	***C. applanatus***
9	Basidiospores with *Qm* < 1.44	***C. chrysanthus***
–	Basidiospores with *Qm* > 1.44	**10**
10	Basidiospores with *Qm* > 1.55	***C. curvatus***
–	Basidiospores with *Qm* < 1.55	**11**
11	Stipe 20–40 × 8–12 mm, basidiospores 7–9 × 5–6 μm, basidia 48–70 × 8–10 μm	***C. aurantinus***
–	Stipe 10–28 × 2–10 mm, basidiospores 6.1–8.1 × 4.1–5.7 μm, basidia 35–72 × 5–9 μm	***C. armeniacus***

## Discussion

Based on morphological characteristics and multigene phylogenetic analyses, four *Cantharellus* species were identified from the Dabie Mountains in the present study, including two new species (*C. armeniacus* and *C. cremeus*), one species newly recorded from Anhui Province (*C. curvatus*), and one previously reported species from Anhui Province (*C. phloginus*). All species identified belong to either *Cantharellus* subg. *Parvocantharellus* or *Cantharellus* subg. *Cinnabarinus*. Species within *Cantharellus* subg. *Cinnabarinus* are typically characterized by small basidiomata, a brightly colored pileus, thin-walled pileipellis hyphae, and abundant clamp connections (Buyck et al. 2014). The two new species described herein conform to these diagnostic features, aligning well with the morphological circumscription of the subgenus.

Among the 21 species of subg. *Cinnabarinus* accepted prior to this study, the majority exhibit orange, yellow, or red coloration, with only *C. cyphelloides* from Japan possessing white basidiomata (Suhara and Kurogi 2015). *Cantharellus cremeus* represents the second white-colored species described within this subgenus. Despite the similar coloration between these two species, *C. cremeus* differs markedly from *C. cyphelloides* in the degree of hymenophore development, stipe morphology, and basidiospore dimensions (Suhara and Kurogi 2015). Prior to this study, eight yellow-colored species had been reported in subg. *Cinnabarinus*. *Cantharellus armeniacus* is distinguished from all of them by a combination of characters, including pileus surface, host association, stipe context and dimensions, hymenophore color, and basidiospore and basidium size. Consequently, both *C. cremeus* and *C. armeniacus* are morphologically distinct from all other members of the subgenus sharing a similar basidiome color, and each is recognizable by its own combination of characters.

Phylogenetic analyses further corroborated the above morphological observations and clarified the precise position of the two new species within the subgenus. The white-colored *C. cremeus* was sister to the orange-red *C. sinocinnabarinus* and showed a close affinity to the cinnabar-red *C. cinnabarinus* (Schwein.) Schwein., whereas it was distantly related to the white-colored *C. cyphelloides*. The vivid yellow *C. armeniacus* was sister to the white-colored *C. cyphelloides* but was distantly related to other yellow species, consistent with its morphological distinctiveness among the yellow-colored taxa. The two white species are not sister to each other, and *C. armeniacus*, although vivid yellow, is sister to a white-colored species. These relationships show that basidiome color is not consistent with phylogenetic affinity in subg. *Cinnabarinus*. Consequently, multigene phylogenetic analyses not only validate the status of these two taxa as new species but also demonstrate that color alone is unreliable for species delimitation in this subgenus, necessitating identification that integrates both morphological and molecular data.

### Ecological associations between subgenera *Parvocantharellus* and *Cinnabarinus* and *Fagaceae* hosts

In this study, the four *Cantharellus* species collected from the Dabie Mountains were assigned to the subgenera *Cinnabarinus* and *Parvocantharellus* and occurred exclusively in association with trees of the family *Fagaceae*. This consistent co-occurrence suggests a high degree of ecological association between these two subgenera and their hosts. In China, all eight reported species of subg. *Cinnabarinus* are associated with *Fagaceae*, while 12 of the 17 reported species of subg. *Parvocantharellus* exhibit the same association. Globally, 15 of 29 species of subg. *Parvocantharellus* and 14 of 23 species of subg. *Cinnabarinus* are linked to *Fagaceae*.

Globally, *Fagaceae* are primarily distributed in tropical and subtropical regions (Zhou 1999), with only a few taxa extending into temperate zones (Wang and Zhang 1991). This distribution pattern is consistent with the tropical–subtropical predominance and limited temperate occurrence of these two *Cantharellus* subgenera. Southwestern and southern China represent diversity hotspots for both subgenera (Wu et al. 2016; Zhang et al. 2025), harboring 14 and 11 known species (both subgenera combined), respectively. These regions coincide precisely with the areas of highest *Fagaceae* richness in China (Qiu et al. 2021, 2024). Progressing northward from these centers, the number of species in both subgenera gradually decreases, paralleling the corresponding decline in *Fagaceae* diversity. Therefore, a significant spatial correlation exists between *Fagaceae* diversity and the species richness of the *Cantharellus* subgenera *Parvocantharellus* and *Cinnabarinus* in China. Collectively, these lines of evidence suggest a close ecological association between these *Cantharellus* subgenera and the plant family *Fagaceae*.

Moreover, subg. *Parvocantharellus* (to which the new record *C. curvatus* belongs) is morphologically similar to subg. *Cinnabarinus* in key aspects, including small basidiomata, abundant clamp connections, and thin-walled terminal hyphae (Buyck et al. 2014). The high degree of overlap in these diagnostic features results in ambiguous boundaries between the two subgenera, highlighting the limitations of relying solely on morphology for delimitation. Furthermore, the traditional view of the monophyly of subg. *Parvocantharellus* has been increasingly challenged by newly discovered species (Buyck et al. 2014), with recent studies suggesting that it may be para- or polyphyletic (Zhang et al. 2022). Specifically, sect. *Cyanomaculati* Buyck & V. Hofstetter (primarily comprising tropical African species) exhibits unstable phylogenetic placement and corresponds well with the morphologically defined subgenus *Afrogomphus* Eyssartier & Buyck. This suggests that this group may represent an independent evolutionary lineage, warranting further taxonomic clarification (Zhang et al. 2021, 2022).

## Supplementary Material

XML Treatment for Cantharellus cremeus

XML Treatment for Cantharellus armeniacus

XML Treatment for Cantharellus phloginus

XML Treatment for Cantharellus curvatus

## Additional information

### Artificial Intelligence (AI) use

The authors accept full responsibility for the content of the manuscript, including the disclosure of any use of AI.

No AI tools were used in the preparation of this manuscript.

### Author ORCIDs

H Chu https://orcid.org/0009-0003-0345-1851

Y.-l. Tuo https://orcid.org/0000-0001-6019-1038

B. Zhang https://orcid.org/0000-0001-9508-8188

### Data availability

All of the data that support the findings of this study are available in the main text.
